# Leukocytospermia and/or Bacteriospermia: Impact on Male Infertility

**DOI:** 10.3390/jcm13102841

**Published:** 2024-05-11

**Authors:** Ralf Henkel

**Affiliations:** 1 LogixX Pharma Ltd., Merlin House, Brunel Road, Theale, Reading RG7 4AB, UK; ralf.henkel@logixxpharma.com; 2 Department of Metabolism, Digestion and Reproduction, Imperial College London, London W12 0HS, UK; 3 Department of Medical Bioscience, University of the Western Cape, Bellville 7535, South Africa

**Keywords:** leukocytospermia, bacteriospermia, oxidative stress, pro-inflammatory cytokines, semen culture, PCR, next-generation sequencing, Endtz test

## Abstract

Infertility is a globally underestimated public health concern affecting almost 190 million people, i.e., about 17.5% of people during their lifetime, while the prevalence of male factor infertility is about 7%. Among numerous other causes, the prevalence of male genital tract infections reportedly ranges between 10% and 35%. Leukocytospermia is found in 30% of infertile men and up to 20% in fertile men. Bacterial infections cause an inflammatory response attracting leukocytes, which produce reactive oxygen species (ROS) and release cytokines, both of which can cause damage to sperm, rendering them dysfunctional. Although leukocytospermia and bacteriospermia are both clinical conditions that can negatively affect male fertility, there is still debate about their impact on assisted reproduction outcomes and management. According to World Health Organization (WHO) guidelines, leukocytes should be determined by means of the Endtz test or with monoclonal antibodies against CD15, CD68 or CD22. The cut-off value proposed by the WHO is 1 × 10^6^ peroxidase-positive cells/mL. For bacteria, Gram staining and semen culture are regarded as the “gold standard”, while modern techniques such as PCR and next-generation sequencing (NGS) are allowing clinicians to detect a wider range of pathogens. Whereas the WHO manual does not specify a specific value as a cut-off for bacterial contamination, several studies consider semen samples with more than 10^3^ colony-forming units (cfu)/mL as bacteriospermic. The pathogenic mechanisms leading to sperm dysfunction include direct interaction of bacteria with the male germ cells, bacterial release of spermatotoxic substances, induction of pro-inflammatory cytokines and ROS, all of which lead to oxidative stress. Clinically, bacterial infections, including “silent” infections, are treatable, with antibiotics being the treatment of choice. Yet, non-steroidal antiphlogistics or antioxidants should also be considered to alleviate inflammatory lesions and improve semen quality. In an assisted reproduction set up, sperm separation techniques significantly reduce the bacterial load in the semen. Nonetheless, contamination of the semen sample with skin commensals should be prevented by applying relevant hygiene techniques. In patients where leukocytospermia is detected, the causes (e.g. infection, inflammation, varicocele, smoking, etc.) of the leukocyte infiltration have to be identified and addressed with antibiotics, anti-inflammatories or antioxidants in cases where high oxidative stress levels are detected. However, no specific strategy is available for the management of leukocytospermia. Therefore, the relationship between bacteriospermia and leukocytospermia as well as their specific impact on functional sperm parameters and reproductive outcome variables such as fertilization or clinical pregnancy must be further investigated. The aim of this narrative review is to provide an update on the current knowledge on leukocytospermia and bacteriospermia and their impact on male fertility.

## 1. Introduction

Infertility is a globally under-recognized public health issue [1], with constantly increasing numbers of couples [2,3], reportedly reaching an estimate of almost 190 million people, struggling to conceive [4,5], which is roughly 17.5% of people during their lifetime [6]. While the percentage of couples where the infertility is only due to a male factor is about 20–30%, the general male contribution to couple infertility is about 50% [7]. Overall, the prevalence of male infertility is about 7% of the male population [8], with the prevalence number continuously increasing by 76.9% between 1990 and 2019, particularly in middle- to high-income countries [9]. The reasons for this dramatic decline in human fecundity are manifold and include socio-economic and lifestyle changes [10,11] as well as environmental pollution [12]. Among the various medical conditions causing male infertility, genital tract infections have a prevalence between 10% and 20%, amounting for up to 35% among a large group of more than 4000 patients attending an andrological outpatient clinic [13]. On the other hand, genital tract infections are conditions that are potentially correctable [14].

Genital tract infection and inflammation go together with an infiltration of the male genital tract with bacteria and leukocytes and can cause serious damage to spermatogenesis in the testes and sperm transit through the genital tract, affecting the functionality of the male accessory sex gland [14,15,16]. Depending on the pathogen, the damage includes impairment of the blood–testis barrier [17], inflammation with dysfunction of the accessory sex glands, hormonal imbalances [18,19,20] or sperm agglutination [21] and impaired sperm functions [22]. The action of pathogens can either be direct by direct interference of bacteria with the male germ cells [23,24,25,26] or indirect by triggering an inflammatory response in the genital tract with the stimulation of leukocytes, which release reactive oxygen species (ROS) and cytokines [27,28,29]. Seminal leukocytes are also significantly elevated in patients with varicocele [30], trauma or certain unhealthy lifestyle conditions such as smoking [31,32], or excessive alcohol consumption [33], which also significantly increase seminal leukocyte concentrations.

Historically, it was believed that human semen is not be colonized with bacteria. Therefore, bacteriospermia, which is defined as the presence of bacteria in seminal fluid [34], is thought to negatively impact male fertility. Although there are numerous reports [35,36,37,38,39,40] about the clinical relevance of the bacterial contamination of semen and the fact that a semen sample is considered bacteriospermic if the semen culture shows more than 10^3^ colony-forming units (cfu)/mL [41], the World Health Organization (WHO) does not mention the term in their two latest manuals for semen analysis [42,43], which might be for various reasons. Therefore, the aim of this narrative review is to provide an update on the current knowledge on leukocytospermia and bacteriospermia and their impact on male fertility.

## 2. Male Genital Tract Infections

According to the WHO [44], more than 1.0 million sexually transmitted infections are acquired daily, rendering these infections a huge public health problem. Infections of the male genital tract include infections and inflammations of the male accessory glands (MAGI; male accessory gland infection) [45] and of other parts of the male genital tract (MGTI; male genital tract infections), a term that was introduced because specific infections of the male accessory glands cannot be distinguished from localized infections of other parts of the male genital tract [46]. Additionally, MGTIs are asymptomatic in about half of cases [18,47].

The most frequently found pathogens in the male genital tract, including sexually transmitted infections, are *Chlamydia trachomatis*, *Ureaplasma urealyticum*, *Neisseria gonorrhoeae*, *Mycoplasma hominis*, *Mycoplasma genitalium*, *Staphylococcus aureus*, *Streptococci* or *Enterococcus faecalis* [19,37,44]. *Escherichia coli*, another frequently detected pathogen, causes epididymo-orchitis and prostatitis in 65% to 80% of cases [18]. These, and other pathogens, such as *Treponema pallidum*, *Trypanosoma* spp. and *Schistosoma* spp., or even viruses can cause infections of the whole male genital tract, such as orchitis, epididymitis, prostatitis, vesiculitis, urethritis or balanitis [19], causing infertility; they can even have long-term consequences for male sexual functions [20].

**Figure 1:**
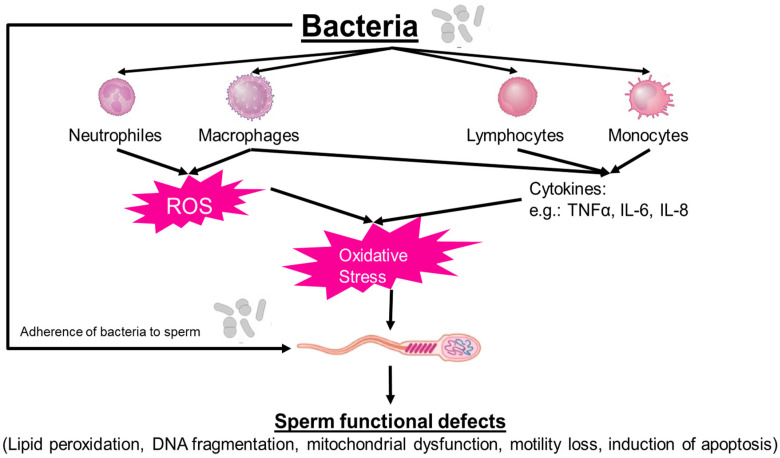
Direct and indirect effects of bacteria on sperm function.

Bacteria affect male fertility potential, not only by causing an inflammatory response in the affected part of the reproductive tract but can also cause significant damage to the sperm by direct interaction with sperm as well as the release of spermatotoxic substances. As a reaction of infections and inflammations, leukocytes infiltrate the affected organs, releasing pro-inflammatory cytokines, like tumor necrosis factor-α (TNF-α), interleukin-6 (IL-6) or IL-8 and ROS [48,49,50]. Bacteria also adhere to the sperm surface by adhesive fibers called ‘pili’, thereby immobilizing sperm and even triggering cell death via apoptosis [51,52]. All this can significantly affect sperm functions and, thereby, male fertility (Figure 1). Bacterial genera, such as *Corynebacterium* or *E. coli*, adhere to cells via pili, thereby affecting sperm motility [53,54]. Others, such as *Enterococcus*, *C. trachomatis* or *Mycoplasma*, are known to form biofilms, a very intricate process [55,56,57], causing the agglutination of sperm with other sperm. According to Monga and Roberts [23], the density and variability of receptors including a sperm agglutination factor (SAF) on the sperm plasma membrane play an essential role for this process [58], thereby affecting fertility outcomes [21]. In *E. coli* and *Staphylococcus aureus*, for instance, sperm immobilization factors were identified [59,60,61]. Apart from direct interaction between bacteria and sperm, bacteria can secrete lipopolysaccharides, hemolysins or quorum-sensing molecules, all of which have shown reproductive toxicity [62,63,64].

## 3. Prevalence of Leukocytospermia

The appearance of leukocytes in the male reproductive tract is a normal physiological feature [65]. Of these, granulocytes appear in human semen, with 50–60% most prevalent frequently, followed by macrophages (20–30%) and T lymphocytes (2–5%) [66]. On the one hand, white blood cells play an important role in immunosurveillance and the elimination of pathogens [67]. On the other hand, however, some authors also discuss the positive role of leukocytes in eliminating dysfunctional and dead sperm [68,69]. Yet, if the seminal leukocyte concentration exceeds 1 × 10^6^ peroxidase-positive cells/mL, as determined with the Endtz test [70], this condition is regarded as leukocytospermia [43], as abnormally high leukocyte concentrations are regarded as indicative of an infection or inflammatory processes [71], and it has been shown to have diagnostic value [72]. Although leukocytospermia is diagnosed in about 30% of infertile men and is found more frequently in infertile than in fertile men [73], with averages between 10% and 20% [74,75], no infection is detected in 80% of these leukocytospermic men [76]. On the other hand, there is no general agreement on the cut-off value, as some authors suggest that the cut-off of 1 × 10^6^ peroxidase-positive cells/mL is too high [77,78,79], while others consider this value too low [66]. Nevertheless, although leukocytospermia is considered an inflammatory disease, which is caused by a bacterial urogenital infection, other studies suggest that leukocytospermia does not necessarily indicate poor sperm functions [29]. Kaleli and co-workers even suggest a favorable role of seminal leukocytes at concentrations between 10^6^ and 3 × 10^6^/mL [69]. This discrepancy could be because of the heterogeneity of the leukocyte population [80] and that only activated leukocytes would exert detrimental effects [79,81].

## 4. Prevalence of Bacteriospermia

Historically, it was believed that human semen is not colonized with bacteria. Therefore, bacteriospermia, which is defined as the presence of bacteria in seminal fluid, is thought to negatively impact male fertility [34]. However, it has been shown that ejaculate is normally populated by commensals such as *Staphylococcus epidermidis* or *S. viridans* and/or contaminated with bacteria of the anterior urethra [82,83]. Although there are numerous reports [35,36,37,38,39,40] about the clinical relevance of the bacterial contamination of semen and the fact that a semen sample is considered “significant” bacteriospermic if the semen culture shows more than 10^3^ colony-forming units (cfu)/mL [41,84], the WHO does not mention the term in their two latest manuals for semen analysis [42,43], which might be for various reasons. On the other hand, *E. coli* and E. faecalis are regarded as pathogens and causes of about 90% of chronic bacterial prostatitis cases (NIH II) [41,85].

Cottell and co-workers reported that about 70% of semen samples are contaminated with non-pathogenic bacteria, which does not mean that this does not necessarily represent an infection [82]. Jarvi et al., using the PCR technique, detected more than 10^4^ bacteria/mL in an equal percentage of infertile and fertile men [86], whereas they detected bacteriospermia using the standard semen culture in only 27% of the infertile men and none in the fertile group. Another study, conducted with 97 healthy men [87], found bacteria in 83% of the semen samples. Different parts of the male reproductive tract have their own specific microbiome, which even varies between different individuals and is influenced by factors, such as diet, BMI, ethnicity, lifestyle habits, hygiene or sexual activity [88].

**Figure 2:**
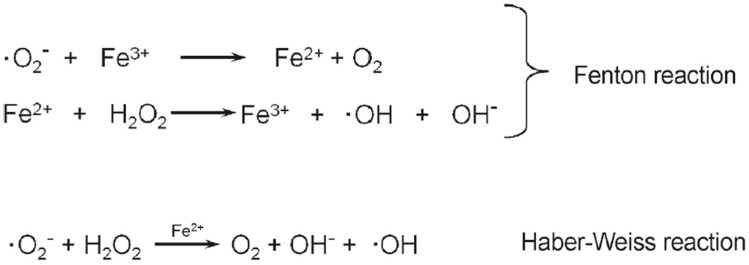
Fenton reaction and Haber–Weiss reaction (from [102]).

Cumming and Carrell reported a positive semen culture in only 12.8% of leukocytospermic patients [89]. On the other hand, it appears that the standard techniques to detect bacteria in semen, semen culture or PCR, are not suitable to evaluate the seminal microbiome, as these techniques do not detect all bacteria [90]. It appears that under- or over-representation of certain bacteria is associated with sperm parameters [91] and that seminal and vaginal microbiomes are associated with *Gardnerella vaginalis*, being predominant in 50% of women where the partners were leukocytospermic, while this was the case in only 5.9% women with non-leukocytospermic partners [92]. In an in vitro model, pretreatment of sperm with probiotic bacteria, *Lactobacillus brevis*, *L. salivarius*, and *L. plantarum*, has been shown to have positive effects on sperm motility and viability and also significantly reduce lipid peroxidation, as determined by BODIPY C_11_ fluorescence after a short-time (20 min) exposure of sperm to an approximately 40-times higher Fe^2+^ concentration than physiological levels to induce lipid peroxidation [93]. Iron ions are known to stimulate the Fenton and Haber–Weiss reactions (Figure 2) to produce hydroxyl radicals responsible for the initiation of lipid peroxidation [94]. Yet, it has also been shown that hydroxyl radicals are not produced in human sperm when lipid peroxidation is promoted by Fe^2+^ [95]. Hence, in this experimental setup, where sperm and bacteria were separated, the protective effect appears to be mediated by soluble antioxidants secreted by the bacteria. On the other hand, these supraphysiological experimental Fe^2+^ levels cannot be directly compared with the physiological situation where Lactobacilli rather protect against ROS when the bacteria colonize mucosal surfaces [96,97]. More recent studies confirmed the positive effects of other probiotic bacteria on sperm parameters as well as on testicular weight and FSH, LH and testosterone concentrations [98,99,100,101]. Hence, it appears that one has to look at “bacteriospermia” in a differentiated way by distinguishing between beneficial and pathological bacteria.

## 5. Quantification of Leukocytes in Semen

Due to the potential detrimental impact of leukocytes on sperm functions and male fertility, leukocytes in semen should be assessed. The latest WHO manual recommends the assessment of seminal leukocytes within 30 to 60 min after ejaculation if required in the section ‘basic semen examination’ [43]. To find out if other cells (epithelial cells or “round cells” (leukocytes and immature germ cells)) than sperm are present in an ejaculate, this should be carried out under high magnification. Like the Endtz test [70], this histochemical technique detects cells that contain peroxidase, namely granulocytes, while it does not detect cells, such as lymphocytes, macrophages or monocytes, that do not contain this enzyme. For the assessment of leukocytes, the WHO recommends staining using o-toluidine. Peroxidase-positive cells will then stain brown, with values of more than or equal to 1 × 10^6^ peroxidase-positive cells/mL being regarded as too high.

Alternatively to the peroxidase stain, the WHO recommends immunocytochemical staining using anti-CD45 antibodies, a marker that detects all leukocytes. In contrast to the peroxidase stain method, there are currently no reference values in human semen for this immunocytochemical method available [43]. Although the WHO only suggests the immunocytochemical methodology of this test, it can also be performed using fluorescence immunological techniques. Apart from CD45 for the general detection of leukocytes, monoclonal antibodies against CD15, CD68 and CD22 can also be used for the specific detection of granulocytes, macrophages and B lymphocytes, respectively. Ricci et al. reported highly significant positive correlations between the number of peroxidase-positive and CD45- and CD53-positive cells in semen [103]. Villegas et al. compared the CD45, CD15 and CD68 immunocytological methods for the detection of leukocytes in human semen with the standard peroxidase stain and the microscopic determination of round cells [104]. While the number of detected cells for the peroxidase and CD68 methods did not differ, significantly higher cell numbers were detected when using anti-CD45 and anti-CD15.

Although the immunocytological methods are highly specific and are, therefore, regarded as “gold standard”, not every laboratory can perform these more time-consuming and expensive tests. Therefore, the WHO recommends the peroxidase test. The American Society for Reproductive Medicine (ASRM) and the American Urological Association (AUA) recommend immunohistochemical tests to confirm leukocytospermia [72].

## 6. Detection of Bacteriospermia

If a bacterial infection is suspected due to physical symptoms, anamnesis and/or poor semen parameters, a rapid and accurate identification of bacterial infection is crucial for therapeutic success. Conventional techniques, such as microscopic evaluation, Gram staining and semen culture, are regarded as the “gold standard” and are accurate and relatively cost-effective [105]. However, these techniques are limited by the possible non-specific biochemical activity of microorganisms or closely related bacterial species as well as the time they take until a result is available, which can take between 48 h for normally growing bacteria and a few weeks for slow-growing bacteria [106]. In addition, some pathogens cannot even be detected as they do not grow under standard conditions. Therefore, polymerase chain reaction (PCR) techniques have been introduced to identify bacterial pathogens, with real-time quantitative PCR (qPCR) providing results faster. More recently, NGS techniques allowing for the analysis and identification of the seminal microbiome have been introduced [107,108]. In comparison to standard PCR techniques, which mainly identify known pathogens, advanced NGS is able to elucidate the whole seminal microbiome with higher accuracy, thereby providing better insight into its composition and interactions. Yet, the latest methods are not readily available in every country and every laboratory and are also more expensive. Furthermore, clinical reference values are not available.

### Comparison of Traditional Semen Cultures versus Microbiological Findings in Semen by PCR Tests

Since male genital tract infections are often asymptomatic, they are difficult to detect [109], and clinicians rely on the presence of leukocytospermia, a history of urethral discharge [110,111] and poor semen quality, especially poor sperm motility, as this may be linked to an infection [112]. This situation is even more problematic as there are no clear clinical guidelines for the indication of a semen culture, as neither the WHO nor the AUA provide clear statements [113]. This might be due to the fact that a positive semen culture is not indicative of male infertility [113], despite indications that leukocytospermic men with asymptomatic infections might benefit from performing a semen culture [114]. Hence, the clinical significance of a positive semen culture continues to be doubtful [115,116]. Although the AUA recommends further testing to differentiate immature germ cells from leukocytes in case an elevated (>1 × 10^6^ round cells/mL) concentration of round cells is detected in the semen analysis, the guideline indicates that routine semen culture has no prospective benefit for infertile couples [117] as there is no clear association between the incidence of leukocytospermia and reproductive success [118]. In contrast, the European Association of Urology (EAU) clearly recommends semen culture or PCR analysis if more than 1 × 10^6^ peroxidase-positive cells/mL were identified after the exclusion of a urinary tract infection [119]. Yet, following EAU guidelines, Ventimiglia and co-workers reported in their cross-sectional study, including 523 asymptomatic infertile men, that among the 54 men showing a positive semen culture, 80% (=43 men) had no leukocytospermia and would have not been diagnosed correctly if they had relied on the EAU guidelines [120]. Moreover, in 120 of the 131 leukocytospermic men, the semen culture was negative and led to 92% unnecessary analyses. On the other hand, with the globally increasing trend of antibiotic resistance of pathogens, a proper diagnosis with antibiotic testing is essential [121]. In turn, this will lead to a further delay in treatment as microbial resistance will have to be tested first.

A number of studies indicate positive rates of semen cultures between 6% and 68% [122,123,124]. This wide range of incidence rates is probably due to variations in the methodology in different laboratories, possible contamination by bacteria deriving from the urethra or skin because of the lack of adherence to the standard procedures for the collection of semen specimens [43]. In addition, numerous pathogens, such as *C. trachomatis*, *U. urealyticum* or *M. hominis*, require challenging culture conditions and might not be detected in a standard semen culture but rather using PCR [113,122,125]. Furthermore, standard semen culture only investigates aerobic pathogens, and little is known about anaerobics [121]. However, anaerobic bacteria, of which about 71% are potentially pathogenic, are also found in human ejaculates [126,127,128].

In comparison to culture-based techniques, which rely on the species-specific characteristics of the suspected pathogens, PCR has the advantage of molecular fingerprinting in PCR, based on the molecular sequence of the DNA, and provides better and quicker pathogen identification [129]. Nevertheless, it is also dependent on prior knowledge of the suspected pathogens and is, therefore, not able to detect unexpected microorganisms [129]. Hence, in order to obtain comprehensive insight into the infection, its pathogens and treatment options, which strictly depend on the specific pathogens identified, techniques should be used that are quick, cost-effective and are also able to identify possible drug resistance. After the extensive genotyping of bacteria in recent years, multiplex PCR has been shown to be effective in identifying even multi-drug-resistant pathogens [130,131]. In laboratories that do not have the facility of the most advanced PCR technology, a combination of semen culture and PCR seems reasonable. Nonetheless, to further improve diagnostics, identification of antibiotic resistance and treatment of patients, novel culture-independent diagnostic methods need to be developed [132].

Despite all the advancements in PCR techniques, knowledge of the pathogens causing an infection is still required, and the simultaneous detection of a multitude of different microbes in one step is not possible. Next-generation sequencing (NGS), however, not only allows one to detect different pathogens in a specimen simultaneously but also the detection and identification of fastidious pathogens, including anaerobes and non-culturable bacteria [133]. The NGS technique further has the advantage of obtaining a comprehensive overview of the microbiome colonizing an organ, thus enabling better understanding of the infection and better clinical decisions [134]. In a study including 112 patients from different areas of urology (acute prostatitis, neurogenic bladder, chronic bacterial prostatitis), Mouraviev and McDonald demonstrated the usefulness of this technology as it improved the clinical efficacy, as compared to standard culture and antibiotic resistance testing [135]. Recent studies indicate that these novel technologies, including whole-genome sequencing (WGS) and metagenomic next-generation sequencing (mNGS), are suitable to predict antimicrobial resistance. allow for the direct detection of microorganisms and resistance genes in clinical samples, thus offering a more rapid and comprehensive approach compared to traditional culture-based techniques [136,137]. NGS can track bacterial clones, identify new antibiotic resistance genes, and their genetic carriers, such as plasmids. Therefore, NGS can be particularly useful in clinical settings for predicting resistance in medically relevant microorganisms like *Mycobacterium tuberculosis*, *Staphylococcus aureus*, and *Neisseria gonorrhoeae* [136]. However, despite the promising features of NGS, the methodology still lacks standardization, accuracy and reproducibility, and the NGS technology needs to be further improved before its routine clinical implementation [138]. Therefore, NGS can only be seen as a supplementary tool to conventional phenotypic susceptibility testing [137]. Currently, the NGS methodology predominantly uses the 16S rRNA gene as a target, as this technique is most cost-effective and widely used technique [108]. Nevertheless, it has emerged from recent studies that not all microorganisms found in human semen are necessarily pathogenic, and male fertility depends on whether certain microbial species are over- or under-represented [91]. In the mid-term perspective, however, and with the further development of the NGS technology and the rapidly evolving field, NGS is expected to play an increasingly important role in diagnostic microbiology. Since only a few studies investigating the microbiome in healthy men are available [139,140,141], we need to learn more about the seminal microbiome and its influence on male fertility potential. Table 1 shows the most important bacterial pathogens causing genital tract infections with the relevant diagnostic methods.

## 7. Pathogenesis of Male Genital Tract Infection

### 7.1. Bacteriospermia

The effects of a bacterial infection on sperm can either be direct and isolated by affecting sperm structure and functions or indirect by triggering an inflammatory reaction with the attraction of leukocytes to the infection site to combat the infection by the release of pro-inflammatory cytokines and ROS [146,147,148]. By this mechanism, pathogens stimulate glucose-6-phosphate dehydrogenase, providing nicotinamide adenine dinucleotide phosphate (NADPH) to fuel the subsequent release of ROS. Fraczek and co-workers investigated a total of 101 healthy men, of which 30 showed isolated bacteriospermia, 19 men had isolated leukocytospermia, while 22 had bacteria and leukocytospermia [35]. In the group of isolated bacteriospermia, sperm concentration, normal sperm morphology and viability, as determined by means of the hypo-osmotic swelling test, were significantly lower than in the control group (n = 30). In addition, sperm DNA fragmentation, mitochondrial membrane potential as functional sperm parameters as well as the percentage of apoptotic sperm were significantly deteriorated in patients with bacteriospermia, as was suggested earlier by others, after exposing sperm to pathogenic bacterial strains [149,150,151]. The authors conclude that bacterial infections are mainly involved in intrinsic mitochondria-dependent apoptotic cell death.

A bacterial infection with subsequent inflammation and bacteriospermia can significantly affect male fertility, not only by directly impairing sperm functions but also potentially leading to a deterioration of spermatogenesis and/or obstruction of the efferent seminal ducts [37,152,153,154,155]. Generally, infections can affect all parts of the male genital tract and, if not treated, can ascend the genital tract and infect other parts [156]. Ascending infections are mainly caused by sexually transmitted pathogens such as *C. trachomatis* or *E. coli* [15,84,157]. While for chronic prostatitis, the effects on semen parameters are rather limited, these effects are more dramatic for acute epididymitis, where about 10% of the patients can develop azoospermia and up to 30% oligozoospermia [153]. An insufficiently treated epididymitis can ascend to the testis and cause damage to spermatogenesis in about 60% of cases [158]. Considering the close vicinity of different sections and organs of the male genital tract, infections are prone to ascend to neighboring organs, thus making it very difficult to distinguish between infections localized to specific organs, e.g., isolated epididymitis vs. epididymo-orchitis [159].

Apart from deteriorating spermatogenesis or obstructing seminal ducts, bacteria cause male infertility by directly affecting sperm functions [25] by (i) direct attachment of the bacteria to sperm cells [160], (ii) altering sperm structure [161,162,163], (iii) impairing sperm metabolism [151,164], (iv) decreasing mitochondrial and plasma membrane stability, thereby decreasing sperm motility [150,151,165], (v) triggering inducing inflammation and oxidative stress [63], and (vi) directly inhibiting sperm functions such as acrosomal loss and acrosome reaction [63,166,167]. Furthermore, bacteria secrete toxins or products of the quorum-sensing system such as lipopolysaccharides [62] and N-3-oxoacyl homoserine lactones [63], respectively, that have significant detrimental effects on sperm motility, acrosome reaction and DNA integrity.

### 7.2. Leukocytospermia

**Figure 3:**
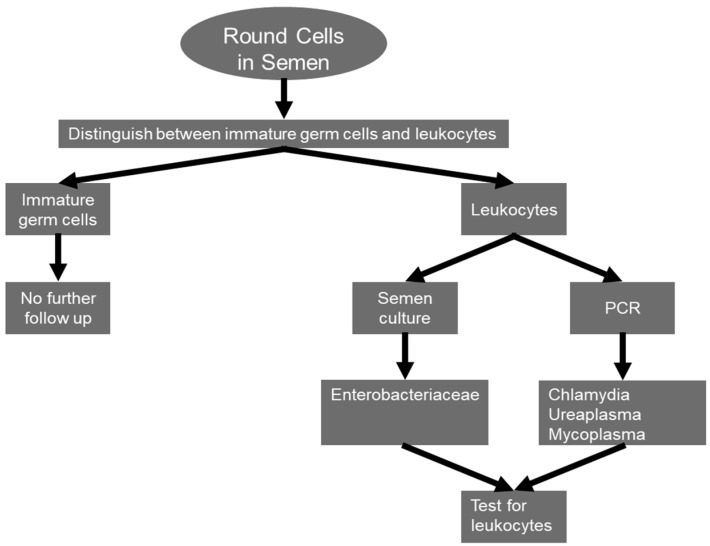
Diagnostic procedure in patients with bacterio-/leukocytospermia (according to [113]).

In their study in 2016, Fraczek et al. investigated 19 patients with isolated leukocytospermia and 22 patients presenting both elevated bacteria and leukocytes [35]. In comparison to the control group, the results in patients with isolated leukocytospermia show significantly reduced sperm concentrations, total and progressive motility, as well as significantly elevated levels of malondialdehyde in sperm lysate as an indication of increased lipid peroxidation. Similarly, in the group of patients with combined bacterio- and leukocytospermia, the sperm concentration was significantly lower. However, in contrast to the leukocytospermia group, sperm vitality, mitochondrial membrane potential, DNA integrity and the percentage of apoptotic sperm were also negatively affected, while the MDA levels in the sperm lysate rather compared to the controls. Generally, the negative effects on sperm functionality in this group (bacterio- plus leukocytospermia) appear to represent a combination of the effects in the isolated groups. While bacteriospermia appears to trigger mitochondria-dependent apoptosis with intracellularly increased ROS levels causing sperm DNA fragmentation, isolated leukocytospermia causes direct oxidative stress by the release of high amounts of ROS by activated leukocytes, causing cellular damage through lipid peroxidation with a more pronounced effect on motility, as reported by Henkel et al. [79]. Hence, there seem to be two different distinct pathogenic mechanisms in isolated bacterio- and leukocytospermia. A diagnostic procedure that should be followed in patients with bacterio-/leukocytospermia is depicted in Figure 3.

### 7.3. Impact of Cytokines on Sperm Function

The main pathogenic mechanisms by which activated seminal leukocytes impair semen quality, thus causing male infertility, are the release of pro-inflammatory cytokines and excessive production of ROS. Various cytokines such as IL-6 or TNFα are normally found in human semen [168]. Yet, their concentrations will be significantly higher in infertile patients or patients with male genital tract infections [49,169] and metabolic syndrome [169]. Significant negative associations between sperm concentration, total and progressive motility, sperm vitality, sperm DNA fragmentation and seminal interleukin (IL-6, IL-8 and TNFα) levels have been described repeatedly [49,170,171], with the highest correlation coefficients observed for the correlations with sperm DNA fragmentation [169]. While the associations for TNFα and IL-8 seems undisputed, there is controversy for IL-6 [49,169]. On the other hand, for TNFα, the associations between the TNFα serum concentration and sperm concentration, total sperm count and sperm DNA fragmentation are even stronger than those for the respective seminal TNFα concentrations [169]. When correlating serum levels of IL-1β, IL-6, IL-8 and TNFα with the respective seminal levels, significant associations were only found for TNFα and IL-6 [25]. It is also noteworthy that, except for TNFα, seminal concentrations of IL-1β, IL-6 and IL-8 were significantly higher than in the serum with median seminal IL8 levels of more than 1200 pg/mL [169]. This is in agreement with the observations of Maegawa and colleagues [172] and Politch et al. [168].

Pro-inflammatory cytokines are also secreted by parts of the male genital tract, such as the epididymis or the prostate [165,173,174], and at physiological levels, they could even be beneficial for the fertilization process by triggering additional ROS production and inducing lipid peroxidation [169]. However, at pathological concentrations, as a result of an infection or inflammation, especially in the presence of leukocytes, the effect to trigger lipid peroxidation seems to be potentiated, thus detrimentally affecting sperm function and male fertility potential. Seshadri et al. [175] reported pathologically high levels of IL-6 in patients with severe oligozoospermia, IL-8 and IL-10 in asthenozoospermic men, whereas IL-6, IL-10 and TNFα are high in patients with obstructive azoospermia. Hence, it appears that the actions of seminal cytokines are dependent on their concentration as well as the interaction of different cytokines. Pro-inflammatory cytokines are toxic for sperm by inducing apoptosis and, thereby, detrimentally affect sperm functionality [116,176,177]. High seminal IL-6 levels can be triggered by leukocytes through elevated ROS production via the nuclear factor kappa-B (NF-κB) [29], a redox-dependent transcription factor [178]. The elevation of ROS production most probably activates NF-κB in leukocytes via the TLR4/NF-κB signaling pathway, thus resulting in an immune and inflammatory response [179].

In this whole context, however, it is also important to note that cytokines and chemokines play significant roles in normal testicular function [180], thus affecting steroidogenesis, spermatogenesis and sperm functions, and more studies need to be conducted to better understand these very complex relationships.

## 8. Leukocytospermia and Seminal Oxidative Stress

Leukocytes are normal components of immunosurveillance in ejaculate [70] and can produce, as part of their physiological function, about 1000-times more ROS than sperm [80,181,182]. However, if the seminal leukocyte concentration exceeds 10^6^/mL, this is considered pathologic, and a number of studies suggest that this condition is associated with poor semen quality and poor results after assisted reproduction [183,184,185]. This is especially the case if these leukocytes are activated [185].

ROS are charged or uncharged highly reactive oxygen derivatives, with half-life times in the nano- (10^−9^ s; •OH; hydroxyl radical) to milli-second (10^−3^ s; •O_2_^−^; superoxide) range and usually react immediately after their production [186]. More stable oxygen intermediates include H_2_O_2_ (hydrogen peroxide), which can even penetrate plasma membranes just like water, and peroxyl (ROO•) and alkoxyl (RO•) radicals with half-life times in the second range (RO•: 7 s). Radicals are molecules or atoms with one or more unpaired electrons in the outer orbit, a feature which renders these molecules chemically highly unstable. ROS are physiologically produced in the mitochondria of all aerobically living cells as a side product of the energy generation in the mitochondrial electron transfer chain. This process is enzymatically controlled oxidative phosphorylation and oxidation of nicotinamide adenine dinucleotide (NADH) as an electron donator, which does not convert the energy of 100% of the consumed oxygen into ATP but converts about 1–5% into free radicals [187,188]. Under physiological conditions, superoxide is dismutated by the enzyme superoxide dismutase into H_2_O_2_, which is essential for cellular redox regulation and cell signaling [189] as it triggers essential physiological functions, including gene regulation, cellular activities or synaptic plasticity [190,191,192,193]. Hence, physiologically, H_2_O_2_ and •O_2_^−^ cannot be regarded as harmful agents but as essential triggers to cellular functions [194]. Therefore, it is critical to compensate these free radicals with antioxidants and maintain the cellular redox homeostasis as high amounts of these radicals will lead to oxidative stress, sperm dysfunction and male infertility [195,196,197]. However, in case of an infection or inflammation, numerous activated leukocytes release high amounts of pro-inflammatory cytokines and ROS, thus shifting the redox homeostasis towards oxidative stress, with its detrimental consequences on sperm plasma membranes and DNA.

**Figure 4:**
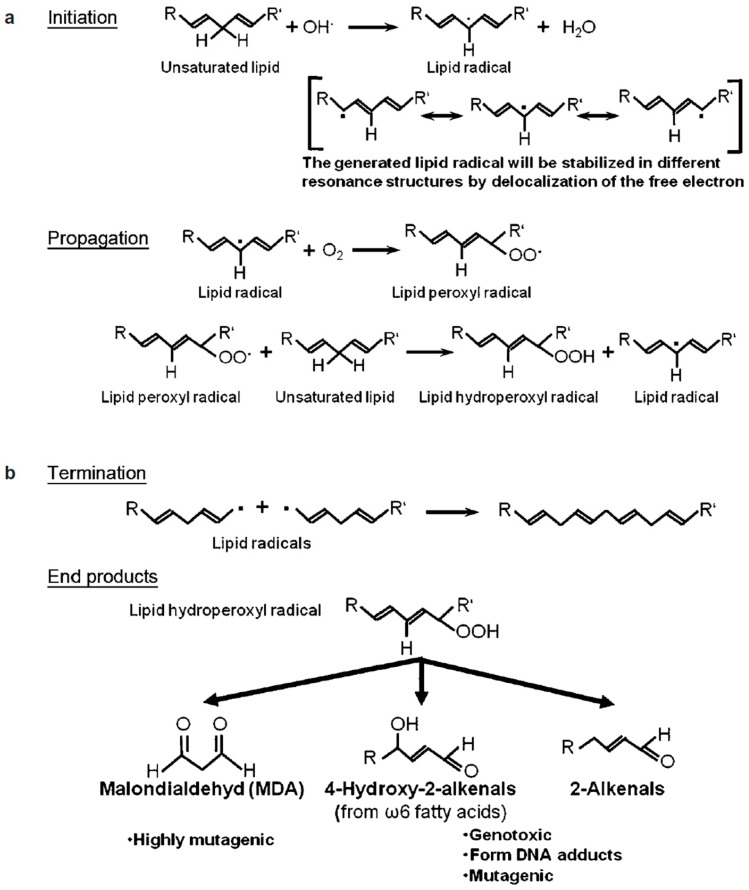
Schematic illustration of lipid peroxidation. (**a**) Initiation and propagation phases. Free radicals react with double bond in the lipids, forming lipid radicals, which react with oxygen to form lipid peroxyl radicals, which in turn react further with neighboring lipids and thereby propagate the reaction in a radical chain reaction. (**b**) When two lipid radicals react with one another, the molecules will be electronically stabilized by forming a stable bond. In the process of lipid peroxidation, lipid hydroperoxyl radicals degrade into various degradation products such as malondialdehyde or 4-hydroxy-2-alkenals (from [102]).

Since sperm plasma membranes contain about 50%, an extraordinarily high amount, of polyunsaturated fatty acids (PUFAs), including docosahexaenoic acid (six double bonds per molecule), arachidonic acid (four double bonds), eicosatrienoic acid (three double bonds) and linoleic acid (two double bonds), contributing about 21%, 2.4%, 2.7% and 4%, respectively, to the total fatty acid of the PUFA content of human sperm [102], human sperm are especially prone to oxidative assaults. If sperm are exposed to oxidative stress conditions, the PUFAs will be oxidized in the process of lipid peroxidation, a process which can be divided into three phases: initiation, propagation and termination (Figure 4). For the initiation and propagation of lipid peroxidation, the initial ROS (•OH and/or •O_2_^−^) are produced by the Fenton reaction and/or the Haber–Weiss reaction (Figure 2), which are catalyzed by transition metal, such as iron or copper. Considering this high sensitivity of sperm plasma membranes to oxidative assaults and the fact that sperm functions are basically membrane functions [198,199,200], lipid peroxidation will decrease the fluidity of sperm plasma membranes and organelle membranes, therefore damaging sperm functions, such as capacitation, acrosome reaction and sperm–oocyte fusion [196], ion gradients and receptor-mediated signal transduction [201]. As end products of lipid peroxidation, a variety of mutagenic and genotoxic degradation products, like malondialdehyde, 4-hydroxy-2-alkenals or 2-alkenals, are formed [202]. Thus, lipid peroxidation not only directly damages the sperm plasma membrane and thereby sperm functions but also indirectly damages the sperm DNA by forming DNA adducts [203,204,205].

On the other hand, excessive ROS also causes direct oxidative damage to sperm nuclear (nDNA) [206,207,208] and mitochondrial DNA (mtDNA) [209,210,211] and has repeatedly been shown to have detrimental impacts on male fertility potential and sperm fertilizing ability. For nDNA, these damages include DNA fragmentation, telomere attrition and epigenetic modifications [212]. The direct actions of free radicals on DNA include reactions with the sugar residues of desoxyribose, a reaction resulting in DNA strand breaks. In case the purine and pyrimidine bases are oxidized, a DNA reading process with a subsequent increased mutation rate will be the result. Furthermore, ROS can cause base modifications, adduct and intra-strand crosslink formation [213]. Direct oxidation of guanine leads to 8-hydroxy-20-deoxyguanosine (8-OHdG), which has been shown to be a useful and specific marker of oxidative DNA damage [214]. It might be possible that the site of 8-OHdG formation could be associated with sites that are poorly condensed and protected by protamines. In addition, oxidative stress triggers mitochondria-related apoptosis by increasing p53 and caspase-3, -6 and -7 following activation of the MAPK pathway [215,216,217].

mtDNA is crucial for a cell’s ATP production as it encodes for 13 proteins of the electron transfer chain, but, in contrast to nDNA, mtDNA is much shorter and replicates much faster than nDNA. Additionally, mtDNA is not protected by histones or protamines, with only very basic repair mechanisms available, and has no proofreading mechanisms [218]. Consequently, mtDNA is reportedly about 100-times more susceptible to oxidative damage, mutations and mitochondrial diseases [219,220]. A loss of mitochondrial function will lead to poor sperm motility [219,221]. Asthenozoospermic patients not only show a significantly increased copy number of mtDNA [222] but also high levels of mutated mtDNA [223]. Defects in the mitochondrial electron transfer chain and elevated MDA levels have been detected in oligoasthenozoospermic patients [211]. Since crosslinks between mtDNA proteins can be formed, this can increase mitochondrial fission and mtDNA damage [224,225], eventually leading to a vicious cycle of ROS production due to damage to the electron transfer chain. Therefore, it is safe to assume that damage to the mitochondria caused by oxidative stress of whatever origin is the major force behind sperm dysfunctions [226].

Telomeres are important non-coding nucleotide sequences (TTAGGG) involved in the maintenance of the genomic organization that have essential roles in maintaining genomic integrity and chromosomal stability [227]. Therefore, shortened telomeres are indicators of aging, cancer and reproductive dysfunctions [228,229,230]. If telomeres are critically shortened, chromosomes become unprotected, and cells are destined to age [231]. Since telomeres are rich in guanine, they are easy targets of oxidative assaults [232,233] and caused by age, smoking, diet, environmental factors or infections [234]. Oxidative stress is regarded as the most frequent mechanism of telomere attrition [235]. In infertile men, significantly shortened telomeres have been found and are associated with oxidative stress, poor sperm concentration, motility, vitality, normal sperm morphology and sperm DNA fragmentation [236,237,238,239]. On the other hand, mild oxidative stress conditions appear to cause telomere elongation [240]. Although the telomere lengths of sperm and leukocytes are positively associated [239], to the best of the author’s knowledge, there is no report published directly linking leukocytospermia or infection-caused oxidative stress to telomere attrition; yet, a link between leukocytospermia or seminal tract infection and telomere shortening appears plausible.

Another way that infections and leukocytes could exert their detrimental effects on sperm functions and, thus, on male fertility is through epigenetic modifications, which can be induced by oxidative stress. In turn, epigenetic modifications can affect gene expression and, consequently, exacerbate reproductive and sperm functions [241,242]. This could be particularly possible in cases of silent genital tract infections. Yet, this topic needs further investigation.

## 9. Relevance of Bacteriospermia/Leukocytospermia in Assisted Reproductive Technology (ART)

### 9.1. Relevance of Bacteriospermia

Although there are no guidelines from the WHO, the AUA, the EUA or the ESHRE on semen culture, it is performed within the diagnostic work-up of male infertility and possible subsequent infertility treatment with IVF or ICSI. However, there is no consensus about its significance and usefulness, and only a few studies have reported the impact of seminal bacterial infections on ART outcomes. Nevertheless, bacteria in semen can potentially impact semen quality and subsequent ART outcomes, and it is, therefore, crucial to identify and appropriately manage any infections prior to ART procedures. Since bacteria are reported to exert detrimental effects on sperm, which include significantly decreased motility, morphology and DNA integrity [243], bacterial infections can have adverse effects on reproductive outcomes. The effects can be either direct via the release of bacterial toxins such as lipopolysaccharides or hemolysins [62,64] and adhesion to the male germ cells, thereby immobilizing and damaging the sperm via pili [54], or indirectly by causing an immunologic response by leukocytes with a release of pro-inflammatory cytokines and ROS [48,49,50].

Moretti et al. examined a total of 1256 patients, including 20 fertile controls [116]. One hundred and seventy-one patients were excluded from further analysis because of hormonal disbalances, varicocele, cryptorchidism, altered karyotype, a previous or ongoing treatment for fertility disorders and sperm defects due to genetic origin. Out of the remaining 1085 patients that were taken into consideration, 417 (38.4%) showed seminal bacteria. Out of the 226 patients with bacteria in their semen, 164 (72.6%) were infertile. Among the 20 fertile controls, none of the patients had bacteria in their semen. Hence, the authors concluded that bacterial contamination is associated with infertility. Yet, Moretti et al. indicate that the analysis of only aerobic and facultative anaerobic bacteria is a limitation of their study because ejaculates are not routinely examined for anaerobes [116]. Eggert-Kruse et al. identified potentially pathogenic anaerobes in 71% of the patients analyzed [126]. In a study comparing 29 bacteriospermic with 55 non-bacteriospermic patients in an ART program, Zeyad et al. reported significantly higher sperm DNA fragmentation [38], higher protamine deficiency as well as lower semen parameter fertilization rates in bacteriospermic patients, as compared to non-bacteriospermic patients after ICSI. Although the number of good-quality embryos and the pregnancy rate in the bacteriospermic group were lower, the difference between the two groups of patients was not significant. However, by increasing the number of patients from 84 to 106, the difference for the good-quality embryos might become significant. These results by Zeyad et al. confirm a previous report by Loutradi et al. [244].

In a study investigating 285 infertile couples, Ricci and co-workers found that 85.7% of patients with successful IVF showed no microbiological contamination, whereas the IVF procedure was successful in only 7.5% of couples with seminal infections with *Enterococcus faecalis*, *Ureaplasma urealyticum* and/or *Mycoplasma hominis* [125]. In a more recent study that investigated the seminal microbiome of normozoospermic men by means of pyrosequencing and real-time quantitative PCR, Štšepetova et al. reported that the presence of *Staphylococcus* sp. and *Alphaproteobacteria* was negatively correlated with sperm motility and embryo quality [245]. *Staphylococcus* sp. was only detected in patients with seminal tract inflammations. On the other hand, Amato et al. could not find any difference in the seminal microbiome between patients who failed to achieve pregnancy after intrauterine insemination (IUI) and those who were successful [246]. However, when employing any ART technique, one must also consider that sperm preparation techniques such as density gradient centrifugation or microfluidics significantly and effectively decrease the bacterial load and select the most functional sperm [244,247]. Therefore, a distinct association between a seminal bacterial infection and successful ART outcome might not be obvious.

Nevertheless, *Chlamydia trachomatis*, *Staphylococcus* sp., *Ureaplasma urealyticum*, *Mycoplasms*, *Escherichia coli* and Gram-positive bacteria such as *Enterococcus faecalis* as potentially pathogenic bacteria should be considered before recommending a patient for assisted reproduction. Yet, it must be stressed that recent studies indicate that ART does not occur in a sterile environment as bacterial species such as *Lactobacillus iners* or *Acinetobacter* had higher ART success rates [91,248].

### 9.2. Relevance of Leukocytospermia

Although leukocytospermia is widely regarded as a negative prognostic parameter for the success in an ART program and the WHO and ESHRE provide guidelines for good practice in ART laboratories [43,72,249], specific guidelines on leukocytospermia and how to manage this condition in the context of IVF or ICSI are not explicitly available. There is a number of studies demonstrating significantly reduced semen quality in cases with leukocytospermia as compared to non-leukocytospermic samples [75,79,183,250]. On the contrary, Kiessling et al., in a small study of 24 patients, of which 11 were leukocytospermic and 13 non-leukocytospermic, reported significantly better normal sperm morphology in the leukocytospermic samples [67]. Looking at the impact of leukocytospermia on the clinical outcomes after ART, only one study demonstrated significantly higher cleavage and clinical pregnancy rates in patients where the male partners were leukocytospermic, while no difference was observed for the good-quality embryo rate [251]. On the contrary, Yilmaz et al. found a negative impact of leukocytospermia on fertilization and embryo development rates after ICSI [183]. Yet, other reports demonstrated that the presence of more than 10^6^ leukocytes/mL in the ejaculate that were used for insemination has no effect on the outcome of assisted reproduction by IVF and ICSI [252,253,254,255]. In a meta-analysis including 254 leukocytospermic and 3613 non-leukocytospermic patients in six and five studies, respectively, Castellini et al. did not observe an effect of the presence of an excessive number of leukocytes on the fertilization rate after IVF or ICSI outcomes (six studies) [256]. However, the overall odds for clinical pregnancy were significantly lower for leukocytospermic patients (five studies).

What is noticeable when analyzing the available data is that there is a clear effect of high leukocyte counts on the ejaculate quality but not on the clinical pregnancy rate. For the interpretation of these results, one also has to take the small number of studies that are available into account. This is most probably due to the different procedures involved. While for the analysis of sperm functions such as motility or DNA fragmentation, the sperm samples are directly processed for the relevant analytical technique, namely microscopical or CASA analysis and TUNEL assay or sperm chromatin structure assay (SCSA), respectively, semen samples are processed and the most functional sperm are selected prior to their use for insemination in any ART technique [72,252]. Hence, despite leukocytes producing cytokines and ROS, which have significant detrimental effects on sperm, sperm separation techniques effectively reduce the number of dysfunctional sperm in the separation process, thereby enriching the percentage of functional sperm and increasing the prospects of successful ART. Therefore, leukocytospermia might be a lesser concern for the embryologist when inseminating oocytes. However, finding ‘leukocytospermia’ should prompt general practitioners, gynecologists, obstetricians and embryologists to refer these patients to an andrologist or a reproductive urologist because elevated leukocyte counts may be indicative of a genital tract infection or inflammation [257]. Even if there is no infection, leukocyte counts can be elevated in patients with varicocele [30,258,259]; thereby, the mechanism is thought to be due to the inflammation of the seminal vesicles, which may be common in male infertility caused by varicocele [259]. Additionally, significantly increased numbers of seminal leukocytes are also found in smokers [32].

## 10. Clinical Management of Bacteriospermia

Since bacterial infections are potentially treatable causes of male infertility, bacteriospermia should be treated. However, in order to obtain optimum treatment results, a number of issues such as “silent”, i.e., asymptomatic, presentations [109], antibiotic resistance [260,261,262], reluctance of patients to talk openly about infertility [263,264] and genital tract infections [265], as well as poor compliance [266], need to be considered, especially in men.

With regard to the high probability of silent genital tract infections, physicians need to consider that asymptomatic leukocytospermia may be indicative of a male genital tract infection [114] and should prompt proper bacteriological testing, including antibiotic resistance [121]. Treatment, however, strictly depends on the type of pathogen, with the first choice of therapy being antibiotics to eradicate the pathogens and normalize ROS and inflammatory parameters. It needs to be stressed that both partners have to be tested and treated, as many of these pathogens are sexually transmitted [19,121]. The type of treatment also depends on the location of the infection. While for cystitis treatment with trimethoprim-sulphamethoxazole or sometimes fluoroquinolones is recommended, for epididymo-orchitis, intramuscular ceftriaxone together with the oral administration of doxycycline is commonly recommended. For non-gonococci bacteria and *Chlamydia* sp., ofloxacin or doxycycline can be taken [267], and for the treatment of urosepsis, broadband antibiotics, such as cefotaxime, ceftazidime, piperacillin/tazobactam, ceftolozane/tazobactam, ceftazidime/avibactam, imipenem/cilastatin and meropenem, are recommended [268].

Since the treatment of acute and chronic prostatitis is problematic because only a few antibiotics penetrate the prostate and its secretions, modern regimens with fluoroquinolones have been recommended [269,270]; an antibiotic treatment of these cases is obligatory. Yet, in patients with inflammatory chronic pelvic pain syndrome, this is rather questionable [271], and therapy with corticosteroids, non-steroidal antiphlogistics or antioxidants should be considered to alleviate the inflammatory lesions and improve semen quality [272,273,274,275]. Antioxidants would scavenge the ROS levels and, thereby, reduce the damage to sperm caused by oxidative stress. Notwithstanding, the effects of antioxidants to improve male fertility potential are still a matter of debate [276,277,278]. Even using the antibiotic options of herbal medicine, such as *Zingiber officinale* or *Punica granatum*, infections with *Escherichia coli* can be successfully treated. Against *Klebsiella pneumoniae* and *Enterococcus faecalis*, *Ocimum sanctum* showed effective antibiotic action [279].

### Prevention of Semen Sample Contamination with Skin Commensals, Urethral and General Bacteria

Previously, semen was considered sterile [86,127]. However, recent reports indicate that it contains specific microbiome, and not all bacteria are pathogenic [91]. In addition, Willén et al. showed that 71% of bacteria colonizing the coronal sulcus are also detected in the distal part of the urethra [87]. It is important that one is able to clearly identify and distinguish potential seminal pathogens from contaminations with skin commensals, urethral and general bacteria, at least for diagnostic purposes and in preparation for assisted reproduction, where these microorganisms may detrimentally affect the success [280]. The latter may be managed using antibiotics [281,282]. Moreover, sperm separation techniques significantly reduce the microbial load [245,247]. On the other hand, in a prospective, controlled study including 93 couples for IVF and ICSI, Krissi et al. found that although a high percentage of the semen samples was contaminated skin commensals [283], and this did not have any effect on the fertilization rate and embryo quality.

Nonetheless, the bacterial contamination during the ejaculate collection should be avoided or at least be minimized. Therefore, the WHO recommends strict hygiene procedures in their laboratory manual [43]. The patients should be provided with clear and unmistakable written and verbal instructions on how to produce a semen sample for analysis that has a minimum risk of contamination with skin commensals. Primarily, the WHO recommends not to use coitus interruptus and condoms because this may lead to incomplete collection and contamination with vaginal fluids, which increases the risk of contamination with bacteria colonizing the penile skin, and agents in the condom may have spermicidal effects [284,285]. Before masturbation, the patient should pass urine, wash his hands and penis thoroughly with soap, rinse the soap properly, dry hands and penis with a clean, preferably disposable towel, and then ejaculate into a sterile wide-mouthed dry plastic container. The patients’ instructions should include information that the container must only be opened just before masturbation and not be touched inside.

Strict hygienic procedures for the semen collection have been reported to decrease the bacterial load [283,286,287,288]. Kim and Goldstein [287] and Rodin et al. [289] even recommended the disinfection of the penile skin, scrotum, buttocks, perianal area and hands with an antibacterial preparation containing 4% chlorhexidine gluconate and 10% povidone-iodine [287]. However, even with all hygienic measures, it is important to note that the specific impact of hygiene practices can vary depending on individual circumstances and the type of bacteria involved. Therefore, a better training of practitioners and wider awareness of patients will certainly assist in minimizing contaminations and result in better diagnoses.

## 11. Clinical Management of Leukocytospermia

Leukocytospermia can be a symptom of systemic and genital tract infections, inflammation and autoimmune conditions, but also varicocele [30] or the consumption of alcohol and drugs as well as smoking [31,32,33] or narrowing urethra or urethral strictures, vasovasostomy and urethroplasty [290], making it a complex and unclear condition. Although the relationship between infection and increased seminal leukocyte concentrations is obvious, this relationship is poor, and the diagnostic value is low [291]. Since there are numerous causes for increased leukocyte counts in semen, it is necessary to identify and treat the underlying cause of leukocytospermia.

Bacterial infections must be treated with antibiotics, as one study showed a significant decrease in seminal leukocyte counts and an improvement in pregnancy rates [292], and four others resulted in a marked, but not significant, improvement in the pregnancy rates [261,293,294,295]. Jung et al., in a systematic review, concluded that antibiotics not only improve semen parameters but also pregnancy rates [68]. In a meta-analysis, Skau and Folstad suggested that treatment of bacterial infections with broad-spectrum antibiotics reduces the leukocyte count in semen and improves the overall quality of ejaculates [296]. Yet, due to the lack of clear evidence of the effectiveness of antibiotic treatments to improve pregnancy rates, Brunner and co-workers suggested increasing the duration of the treatment [72]. Inflammations, on the other hand, can be improved with anti-inflammatory treatments, particularly with Cox-2 inhibitors [71,76,272] or anti-inflammatory diets [297], which can reduce the ROS produced by leukocytes. Even antioxidant therapies with curcumin [298] or quercetin [299] have been reported to be effective. In contrast, for anti-inflammatory drugs, such as diclofenac, negative effects on male fertility have been reported [300]. Given the low availability of high-quality studies on this topic, a combination of antibiotic therapy and frequent ejaculation may be recommended [261,294]. For poor lifestyle habits, lifestyle corrections in terms of cessation of smoking, using drugs or reducing the alcohol intake, must be discussed with patients. In patients with a varicocele, the repair thereof should be recommended, following a recent meta-analysis varicocelectomy significantly improved sperm concentration, total motility, and progressive motility after surgery [301]. Yet, no specific study is available that has investigated the effect of varicocele repair on seminal leukocyte counts.

While treatment of the causes of leukocytospermia should have first priority, the oxidative stress that may arise from activated leukocytes as well as from systemic inflammatory responses due to poor lifestyle choices is often treated with antioxidants. To protect the sperm from oxidative damage, seminal fluid is normally rich in antioxidants, such as vitamins C and E, catalase, glutathione, superoxide dismutase, as well as zinc and selenium [302]. The latter two are co-factors of the antioxidant enzymes superoxide dismutase and glutathione peroxidase, respectively [303,304], with the levels of these micronutrients generally being significantly higher in semen than in blood [305,306]. Together, these antioxidants aid in protecting sperm from oxidative assaults to sperm plasma membranes and DNA. Patient supplementation resulted in positive effects on sperm functions [307,308,309,310,311] as well as in no observed effects [276,312,313]. Similarly, supplementation with zinc as a cofactor for antioxidant metalloproteins, which are essential for prostate, epididymal and testicular function [314] and has also been reported to have antimicrobial activity [315,316], showed positive effects on sperm parameters [317], thus supporting an earlier meta-analysis by Zhao et al. [318]. On the contrary, Jenkins et al. [319] and Schisterman et al. [320] did not find any beneficial effects on life birth outcomes. Recent Cochrane studies indicate that there is only low-quality evidence suggesting a positive effect of antioxidant treatments of the male on clinical pregnancy or live birth rates [277,321]. Reasons for these obvious discrepancies in the effects of these treatments include that the study designs are too different, with different formulations, different antioxidant concentrations, different treatment durations and different outcome parameters of the treatment. such as sperm motility, sperm DNA fragmentation, clinical pregnancy or live birth rates. The specific kind of patients investigated also plays a role, as Rochdi et al. [322] reported the positive effects of antioxidant supplementation in idiopathic oligoasthenoteratozoospermic patients. In a large systematic review and meta-analysis, Agarwal et al. concluded that the failure to demonstrate the effectiveness of antioxidant treatments in improving pregnancy rates is due to the availability of only very few randomized controlled trials [288].

Since no clear strategy for the management of leukocytospermia is available, more studies need to be conducted to better characterize the condition, possible treatment options including the association with infections and inflammations, the effects of lifestyle changes or varicocele repair on the seminal leukocyte count and on reproductive outcome parameters, such as fertilization or clinical pregnancy.

## 12. Conclusions

Although bacterio- and leukocytospermia are clinically described conditions, with evidence of negatively affecting male fertility, there is disagreement about the categorization with cut-off values, the consequences for assisted reproduction as well as the therapeutic treatment options. For bacterial infections, several pathogens cause clinically clearly described conditions, such as prostatitis or epididymitis. Yet, recent research employing advanced techniques shows that several bacteria such as probiotic *Lactobacillus* spp. in the seminal microbiome can positively influence sperm motility. Furthermore, in view of the inflammatory response with the attraction of leukocytes to the infected area and the release of pro-inflammatory cytokines and ROS, which have a detrimental influence on steroidogenesis, spermatogenesis and sperm functions, more studies need to be conducted to better understand these very complex relationships. Similarly, although sperm separation techniques significantly reduce the number of leukocytes and the impact of leukocytospermia on male fertility, no generally accepted strategy for its management is available. The effects of leukocytes on sperm functions appear to be linked to their activation status. Additional questions that need to be answered revolve around the characterization of the condition, the effects of lifestyle changes or varicocele repair as well as its impact on reproductive outcome parameters, such as fertilization or clinical pregnancy. Hence, while the need for the antibiotic treatment of an infection is obvious, the impact of the microbiome and the impact of leukocytes on male fertility need to be further investigated.

## Figures and Tables

**Table 1:** Some bacterial pathogens causing genital tract infections.

Pathogen	Disease	Diagnostic Test	Reference
*Chlamydia* *trachomatis*	Urethritis Prostatitis Orchitis Epididymitis	PCR *C. trachomatis* culture with immunofluorescent staining of reticulate bodies	[109,111,113,142,143]
*Ureaplasma* *urealyticum*	Urethritis Prostatitis	PCR Semen culture	[111,113,142]
*Ureaplasma* *parvum*	Urethritis	Semen culture PCR	[113]
*Mycoplasma hominis*	Urethritis	PCR, RT-PCR	[111,113,144]
*Mycoplasma* *genitalium*	Urethritis	PCR	[111]
*Neisseria* *gonorrhea*	Urethritis Orchitis Epididymitis	PCR Gonococcal culture	[143,145]
Gram-positive cocci (e.g., *Enterococcus* spp.)	Prostatitis	Semen culture	[111,113]
Enterobacteriaceae (e.g., *Escherichia coli*, *Klebsiella* spp.)	Urethritis Prostatitis Orchitis Epididymitis	Semen culture API 20E test	[111,113]
